# Establishing Key Domains for Measuring Workplace Mental Health: The Indicators of A Thriving Workplace Survey

**DOI:** 10.1007/s10926-025-10302-6

**Published:** 2025-05-24

**Authors:** Ross Iles, Dianne M. Sheppard

**Affiliations:** 1 SuperFriend, Level 2/157 Spring Street, Melbourne, VIC 3000 Australia; 2 Healthy Working Lives Research Group, Monash University, Melbourne, VIC 3004 Australia; 3 Monash University Accident Research Centre, Monash University, Clayton, VIC 3800 Australia

**Keywords:** Workplace mental health, Factor analysis, Industry, Psychosocial health, Workplace culture

## Abstract

**Purpose:**

The importance, value, and benefits of fostering mentally healthy workplaces are well established. The Indicators of a Thriving Workplace (ITW) questionnaire measures a range of individual psychological and organisational factors as lead indicators of workplace mental health. The current study aimed to develop a valid summary set of indicators or domains of workplace mental health to enable comparisons across industries and sectors nationally.

**Methods:**

Exploratory factor analysis and principal components analysis were sequentially performed on survey data from two independent samples selected from a nationally representative and large (*n* = 9,947) cohort of Australian workers.

**Results:**

Five domains of workplace mental health aligning with the integrated approach to workplace mental health emerged and were confirmed: Leadership, Connectedness, Safety, Work Design, and Capability. When average domain scores were compared across industry, small but statistically significant differences were identified.

**Conclusion:**

The validation of these domains positions the ITW questionnaire as the first comprehensive measure of workplace mental health and well-being that focuses on thriving as a positive construct for Australian workers. Further, industry-based Domain profiling could provide a basis for the prioritisation of efforts to improve workplace mental health, with successful initiatives and practices perhaps adaptable to other industries. Interventions addressing workplace mental health are likely to be more successful when they are industry specific, although interventions responding to mental health challenges in the workplace, such as those tackling mental health-related stigma, may require less cross-industry tailoring.

## Introduction

Approximately 30% of our waking lives are spent in occupational settings, where environmental characteristics, organisational culture, interpersonal dynamics, and management protocols constitute critical determinants of health and well-being [1, 2]. Negative workplace environments, characterised by excessive workload demands, chronic occupational stress, and professional burnout without adequate organisational support mechanisms, have been identified within seven key priority action areas within the National Mental Health Workforce Strategy 2022–2032 [3] and as such, contribute to the determination of mental health as a complex multifactorial construct. The significance of psychologically safe and supportive workplace environments for protecting workers’ health has recently gained legislative recognition in Australia through substantive regulatory amendments that explicitly articulate employers’ duty of care obligations regarding the systematic monitoring and management of hazards and risks to workers’ psychological health and safety [4].

Poor mental health at work negatively impacts productivity and absenteeism and leads to significant costs in terms of sick leave, medical treatment, and insurance-based claims. In Australia, the estimated cost of reduced productivity and workforce participation due to psychological and psychosocial injury is between $12 and $40 billion (AUD) per year, not including the additional costs associated with staff turnover due to mental health challenges [5]. Workers’ compensation claims are increasing with the most recently available data reporting a 37% increase in mental health claims between 2017–2018 and 2021–2022, with anxiety and stress disorders the most commonly reported cause of claim [6, 7]. Workers’ compensation claims for mental health conditions are more than three times as costly and result in more than four times the time off work compared to all other claim types, with 30.7 weeks lost (median) per serious claim [6, 7].

A proactive approach to accurately measuring and supporting the mental health of employees requires robust and evidence-based measures designed to meet the needs of a diverse workforce that are situated within a relevant theoretical framework. In response, the past 15 years has seen a proliferation of tools designed measure various aspects of psychosocial risk in the workplace with the intent to better measure and manage workplace mental health and well-being. An early example is the Australian Workplace Barometer (AWB) tool, developed by Dollard and colleagues in 2009 [8] that is implemented at the workplace level. The AWB comprises scales that assess psychosocial factors that impact well-being, engagement, and effectiveness within organisations using a range of psychometrically validated measures [9]. The *Thriving from Work Questionnaire* was recently developed in the United States (US) designed to measure workplace psychosocial health and well-being nationally [10]. Aligned with the concept of thriving and optimising employee well-being, this comprehensive tool focuses on *positive* work-related well-being across several dimensions and has been recently validated using a sample of workers within the US, however, is contextualised to local health and employment systems. Whilst there are likely to be some similarities between the US and Australian workplaces, differences in population density, industry profile and legislation related to workplace health and safety limit direct application to the Australian context.

Data generated by comprehensive measures capturing nationally representative data relevant to workplace mental health are invaluable for organisations seeking to identify and address psychosocial hazards within comparable workplace contexts, thereby enabling evidence-based prevention strategies and ensuring compliance with Work Health and Safety (WHS) legislation [12]. Moreover, effective and *targeted* communication of those risks unique to organisations of particular scales and industries is essential for effective risk management and translation of research into actionable workplace interventions.

A key difficulty in providing evidence-based and actionable recommendations in this area is the heterogenous nature of industry sectors [11]. How psychosocial hazards present and are likely to be controlled will vary greatly depending on the work being performed. Whilst definitions of negative workplace events and psychosocial hazards tend to be consistent across policy documents [12–15], identifying and addressing bullying in the financial sector, for example, is likely to be practically very different to actions required to do the same in the agriculture industry. Existing strategy documents, such as the Australian Work Health and Safety Strategy, identify priority industries based on compensation claim patterns, including Agriculture, Construction, and Road Transport [4]. Exposure to psychosocial hazards is likely to vary by industry in a similar manner, and the development of tools that identify industry-specific hazard profiles is likely to provide a necessary platform to develop tailored approaches to intervention [11, 16]. It is also possible that the effectiveness of policies already in place to maximise workplace mental health and well-being [17, 18] could be further enhanced when focused on the priority hazards and risks identified for each industry sector.

The importance, value, and benefits of fostering mentally healthy workplaces is well known. The current paper describes the **Indicators of a Thriving Workplace (ITW) questionnaire**, a tool developed to measure a range of individual psychological and organisational factors as lead indicators of workplace mental health at a national and industry level. The questionnaire has been designed to be sensitive to the unique social, economic, and geographical variability across Australia and to provide a window to examine the extent to which industries in Australia are positioned to support employee mental health and well-being. The aim of the current study was to validate a summary set of indicators or domains of workplace mental health to enable comparisons across industries and sectors. These indicators, situated within the integrated approach to workplace mental health conceptual framework [19], facilitate theoretically sound and evidence-informed application of this measurement tool to capture the national status of workplace mental health. The ‘integrated approach to workplace mental health’ [19] was the conceptual framework that underpinned the emergence of the psychosocial workplace domains of the ITW questionnaire. At the core of this approach is the equal emphasis placed on **protecting** mental health and preventing harm, **promoting** mental health by leveraging and maximising the positive aspects of work, and also **transforming information into action** that responds in a targeted fashion to mental health challenges as they arise [19].

A secondary aim was to establish whether there are differences between the emerging Domain scores across industries, with the potential to serve as a basis for industry-specific intervention. This study is of high relevance to the broad discipline of occupational health, including the promotion and sustainability of health and well-being in the workplace setting, prevention of psychological illness and injury, and protection from work-related psychosocial hazards.

## Methods

The ITW survey has been conducted annually since 2015, with incremental changes applied each year. A major review of the ITW survey (consisting of 40 items) was conducted in 2022 to ensure that it aligned with the most contemporary theory and evidence surrounding factors contributing to workplace mental health. The review included a comprehensive literature review, workshops with subject matter experts and examination of existing survey questions that identified and filled any construct measurement gaps relevant to the determination of workplace mental health and well-being. For example, questions related to the equal distribution of resources and access to opportunities were added as measures of organisational fairness. This elicited a refined ITW questionnaire consisting of 199 items, 105 of which addressed workplace mental health and well-being. The original ITW survey established five work-related well-being domains. However, having expanded the number of concept areas and size of data collection through the refined ITW question set, it was important to determine whether domains existed within the data and confirm the contents of emerging domains. The primary focus of this paper is to describe the dimensionality testing and validation of the emergent domains of this refined ITW questionnaire and position any emerging domains within the integrated approach to workplace mental health [19] conceptual framework.

For the purposes of this study, we included only Australian workers due to the uniqueness of our national landscape with respect to cultural, social, political, and economic environments, and employment and labour patterns, all of which can impact organisations and the well-being of their employees [2].

### Data Collection

This study used data from an overall sample of 9,947 Australian workers recruited by Dynata Global, an experienced, external data collection agency. A predetermined sample size was used to permit extensive examination of range of workplace and demographic factors. Australians of working age who were currently employed and working with at least one other person who had already consented to receive research participation invitations from the agency were invited to participate and received a non-cash, points-based incentive for survey completion. The panel from which the respondents were selected is a verified research-only panel with strong quality recruitment measures built in for panellist selection.

Data collection was conducted between 26th October and 22nd December, 2022. Quotas were applied to capture a representative spread of gender, age, geographical location, and industry according to Australian Bureau of Statistics Labour Force data [20] to be representative of the Australian workforce. Once the target sample size was reached, quality control data cleaning processes resulted in the deletion of 307 responses; these included (in order of completion):

- Removal of responses of less than 7 min duration (data thresholds indicated these were “speed completer” responses and were not true considerations of the questions asked);
- Removal of responses that indicated a neutral response bias (using statistical methods to determine the threshold for exclusion); and
- Removal of duplicates (retaining the first response only).

### Survey Instrument

#### Survey Content

The final survey, following its refinement based on formative research (see, e.g. [21]) steps as described above, consisted of 199 items, 105 of which were related to workplace factors, broadly mapped to 22 psychosocial themes or focus areas as determined by the comprehensive literature review (Table 1). Remaining questions addressed demographic information, work characteristics and presence of workplace policies. Participants self-selected the industry they worked in from a list of 19 ANZSIC categories as specified by the Australian Bureau of Statistics (ABS) [22].

**Table 1:** High-level question themes (number of items in parentheses)

Physical environment (3)	Recognition and reward (3)
Learning and career development (3)	Violence (3)
Traumatic events (3)	Bullying (3)
Harassment (3)	Discrimination (3)
Mental health support (5)	Organisational fairness (3)
Change management (7)	Job clarity and control (4)
Job demands (4)	Job security (3)
Interpersonal relationships (14)	Peer support (2)
Managers (11)	Senior leaders (10)
Work–life balance (4)	Work-related fatigue (3)
Feeling safe to speak up (3)	Purpose and meaning (3)
Social impact (5)	TOTAL ITEMS: 105

The item format consisted of a positively worded statement regarding the concept (for example, “At my workplace people have clear job descriptions”) with a five-point Likert response scale from strongly disagree (1) to strongly agree (5).

### Statistical Analyses

Exploratory factor analysis (EFA) was conducted to determine whether suitable summary scales (domains) existed within the data [23]. A two-pronged approach was taken. The first aimed to identify the number of factors best suited to the data. The second aimed to identify the fit of the identified factor solution in line with previous analyses of data collected in this manner. The survey sample was split into two parts. A random selection of 75% of responses were allocated to the development sample, with the remaining allocated to a confirmation sample and a confirmatory factor analysis (CFA) conducted to assess whether the identified factor structure could be verified. This approach was applied to provide the largest development sample possible whilst retaining a representative confirmation sample.

The data were deemed appropriate for principal components analysis (PCA) through confirmation of an adequate number of variables with correlations *r* > 0.3, Bartlett’s test of sphericity reaching statistical significance and Kaiser–Meyer–Olkin (KMO) measure exceeding the minimum value of 0.6 [23, 24]. PCA was used to allow exploration of the most suitable number of factors within the data [24]. Scree plots were investigated and parallel analysis applied to arrive at the best fit solution [25]. Given the large size of both the development and confirmation samples, items were retained throughout this iterative process if component loadings and communalities exceeded 0.30 while not cross-loading on another component > 0.30 [26]. Items not loading on any factor were removed and analysis repeated with the item excluded. To arrive at the final composition of each factor, items with eigenvalues > 0.5 were retained.

A score for each Domain was determined by adding responses from 1 to 5 for each item to arrive at a total score using all survey responses. Scores were transformed to provide a score on a 0–100 scale by applying the formula (average item score −1)*25. Respondent scores from each industry were used to determine the industry average score for each domain. One-way ANOVA was conducted to examine whether differences in average domain scores were significant across industry groups. To reduce probability of Type 1 error, Bonferroni correction was applied to set the significance level at *p* < 0.01 [23]. All analyses were conducted using SPSS software (IBM SPSS Statistics, version 29.0).

## Results

### Participant Demographics

Demographics of the overall, development and confirmation samples are summarised in Table 2. A total of 9,947 people completed the survey. Using SPSS random allocation to partition approximately 75% of cases, 7,447 respondents were allocated to the development group and 2,500 were allocated to the confirmation group. Comparison of proportions in each sample across gender, age, state, and size of workplace characteristics showed the Confirmation sample had a significantly higher proportion of respondents aged between 18 and 24 compared to the Development group (chi square = 5.075, *p* < 0.05). There were no other significant differences between development and confirmation samples.

**Table 2:** Demographic information of survey respondents overall and those for the development and confirmation samples

	Total	Development	Confirmation
*n*	9947	7447	2500
Gender (*n*, % female)	5129 (51.6)	3838 (51.5)	1291 (51.6)
Age (*n*, %)			
18–24	1210 (12.2)	875 (11.7)	335 (13.4)*
25–34	3523 (35.4)	2639 (35.4)	884 (35.4)
45–54	2723 (27.4)	2055 (27.6)	668 (26.7)
55–64	1590 (15.9)	1200 (16.1)	390 (15.6)
50–54	641 (6.4)	479 (6.4)	162 (6.4)
65 +	260 (2.6)	199 (2.7)	61 (2.6)
State (*n*, %)			
New South Wales	3214 (32.3)	2403 (32.3)	811 (32.4)
Victoria	2576 (25.9)	1936 (26.0)	640 (25.6)
Queensland	1736 (17.5)	1292 (17.3)	444 (17.8)
South Australia	736 (7.4)	566 (7.6)	170 (6.8)
Western Australia	1184 (11.9)	886 (11.9)	298 (11.9)
Tasmania	258 (2.6)	188 (2.5)	70 (2.8)
Northern Territory	94 (0.9)	67 (0.9)	27 (1.1)
Australian Capital Territory	137 (1.4)	103 (1.4)	34 (1.4)
Size of workplace			
2–19	1621 (16.3)	1195 (16.0)	426 (17.0)
20–199	2758 (27.7)	2075 (27.9)	683 (27.3)
200–999	2544 (25.6)	1927 (25.9)	617 (24.7)
1,000–4,999	1681 (16.9)	1245 (16.7)	436 (17.4)
5,000 +	1343 (13.5)	1005 (13.5)	338 (13.5)
Industry			
Health Care and Social Assistance	1396 (14.1)	1066 (14.3)	330 (13.2)
Retail Trade	987 (9.9)	752 (10.1)	235 (9.4)
Professional, Scientific, and Technical Services	919 (9.3)	691 (9.3)	228 (9.1)
Construction	896 (9.0)	649 (8.7)	247 (9.9)
Education and Training	837 (8.4)	640 (8.6)	197 (7.9)
Manufacturing	700 (7.0)	531 (7.1)	169 (6.8)
Accommodation and Food Services	681 (6.9)	516 (6.9)	165 (6.6)
Public Administration and Safety	553 (5.6)	420 (5.6)	133 (5.3)
Transport, Postal, and Warehousing	522 (5.3)	379 (5.1)	143 (5.7)
Administrative and Support Services	379 (3.8)	280 (3.8)	99 (4.0)
Financial and Insurance Services	377 (3.8)	275 (3.7)	102 (4.1)
Wholesale Trade	304 (3.1)	227 (3.1)	77 (3.1)
Other Services	264 (2.7)	188 (2.5)	76 (3.0)
Agriculture, Forestry, and Fishing	215 (2.2)	157 (2.1)	58 (2.3)
Arts and Recreation Services	207 (2.1)	157 (2.1)	50 (2.0)
Information Media and Telecommunications	187 (1.9)	138 (1.9)	49 (2.0)
Mining	186 (1.9)	135 (1.8)	51 (2.0)
Rental, Hiring and Real Estate Services	175 (1.8)	129 (1.7)	46 (1.8)
Electricity, Gas, Water, and Waste Services	145 (1.5)	104 (1.4)	41 (1.6)

^*^Significant difference, *p* < 0.05

Initial EFA analyses indicated an 11-factor solution. The purpose of the analysis was to isolate a set of interpretable factors, i.e. which have a simple structure [27] and identify indicators appropriate for industry-specific intervention. Communication and interpretation of more than ten factors were likely to be a barrier to effective application of the resulting analysis. As such, the decision regarding the most appropriate factor solution was based on this requirement for a smaller number of factors combined with statistical indicators of the suitability of each factor [28]. As a result, analysis explored the suitability of a smaller number of factors, including a five factor structure applied to previous ITW surveys. The results of this analysis form the subject of this paper.

PCA of the development sample identified a five factor solution with acceptable reliability coefficients. A total of 47 items were retained, explaining 53.3% of the total data variance (Table 3). The contents of each factor (hereafter referred to as domains) were identified to reflect higher-level constructs described as Leadership, Connectedness, Safety, Work Design, and Capability. The first domain, Leadership, consisted of questions related to managers and how managers conduct their role within the workplace. One item (Managers demonstrate how they support their own mental health) returned an eigenvalue below 0.5 (0.44) but was retained in the analysis based on conceptual fit with questions within the factor [23] and pending confirmation analysis findings. The second domain, Connectedness, contained items centred around the quality of relationships with colleagues in the workplace. The Safety domain centred around harassment, discrimination, and bullying and how these are addressed within the workplace. The Work Design domain consisted of questions about how work roles are conducted and the level of influence people have regarding how, where, and when they work. The final domain, labelled Capability, consisted of seven items focussed on the level of skills and knowledge related to supporting mental health and well-being at work.

**Table 3:** Retained items of each domain and associated component loadings for the development sample

	1	2	3	4	5
	Leadership	Connectedness	Safety	Work Design	Capability
Cronbach’s alpha coefficient	0.933	0.853	0.926	0.915	0.888
Item component loadings					
Managers demonstrate the values of the workplace​	0.59				
Managers advocate for the best interests of their team​	0.58				
Managers support their team to work together​	0.62				
Managers provide constructive feedback​	0.61				
Managers are available when people need them​	0.57				
Managers genuinely consider other people’s ideas	0.60				
Managers match tasks with people’s strengths​	0.56				
Managers help their teams to solve problems​	0.61				
Managers make sure people have the resources they need to do their job well	0.54				
Managers demonstrate how they support their own mental health​	0.44*				
I treat others with respect​		0.63			
I appreciate others whose backgrounds are different from my own​		0.64			
I am honest with others​		0.61			
I feel that I approach situations with an open mind​		0.56			
People care about each other​		0.54			
I trust the people with whom I work​		0.50			
I feel like I am part of a team​		0.51			
I feel that people use appropriate humour​		0.51			
People are polite to each other​		0.53			
I feel that people work together effectively​		0.54			
There are processes to minimise harassment​			0.64		
There is an effective process to investigate reports of harassment​			0.64		
There is a zero tolerance approach to harassment​			0.70		
There are processes to minimise bullying​			0.61		
There is an effective process to investigate reports of bullying​			0.63		
There is a zero tolerance approach to bullying​			0.68		
There are processes to minimise discrimination​			0.62		
There is an effective process to investigate reports of discrimination​			0.62		
There is a zero tolerance approach to discrimination​			0.67		
There is support available if violence is experienced at work​			0.51		
Managers support the practice of good work–life balance​				0.60	
My workplace recognises work-life balance looks different for everyone				0.71	
My workplace supports flexible working arrangements				0.82	
My workplace provides equal access to flexible work arrangements regardless of individual circumstances				0.75	
People at my workplace have enough time to rest between scheduled work times​				0.70	
People at my workplace can work at times that best suit them				0.80	
People at my workplace have enough breaks whilst they’re at work to recharge				0.70	
People at my workplace are given flexibility to control when they complete work tasks				0.70	
People at my workplace are given flexibility to control how they complete work tasks				0.63	
People at my workplace can contribute to decisions that affect their job				0.53	
There is access to confidential counselling or support services​					0.77
There are no barriers to accessing confidential counselling or support service​					0.68
In general, people in my workplace have the skills and expertise to support each other’s mental health​					0.59
In general, people in my workplace have access to mental health education​					0.79
Managers participate in regular mental health training​					0.68
There is support for training initiatives aimed at improving human resilience​					0.55
There is adequate training in how to respond to traumatic events at work​					0.53

^*^Eigenvalues for this item fell below the 0.5 threshold for the development sample and above the threshold for the confirmation sample, leading to its inclusion in the overall domain

The analysis was repeated on the confirmation sample (*n* = 2,500). Factor loadings for the confirmation sample are provided in Appendix A. The Leadership domain was confirmed with 10 items with loading on this factor with eigenvalues greater than 0.5. The item loading below 0.5 in the development sample (Managers demonstrate how they support their own mental health) loaded more strongly in the confirmation sample and was included in the final domain due to strong average loading across both samples and the perceived conceptual fit. The Connectedness domain contained five items that returned eigenvalues lower than 0.5, but remained the strongest loading items for the domain and were retained. The Safety domain was confirmed with all 10 items returning high eigenvalues with no cross-loadings. The Work Design domain was confirmed with one item returning an eigenvalue of 0.48. Whilst three items in the Safety domain fell just below eigenvalues of 0.5, this domain was also confirmed. Overall analysis of the confirmation sample supported the original 5-factor solution and domain contents.

Scores for each industry were calculated for each domain and are presented in Table 4. A series of one-way ANOVAs for each domain revealed statistically significant but relatively small differences between industries in mean scores for each of the domains (*p* < 0.01). There was greatest variation in the scores for Work Design, as demonstrated by the largest partial eta of 0.041, equivalent to a small-medium effect size [29]. The differences in the Leadership and Capability domains were similar and equivalent to a small size effect [29]. Connectedness showed the least variation across scores, with observed differences likely to be very small in this domain.

**Table 4:** Industry domain scores and ANOVA results [4]

Industry	n	Scores									
		Leadership		Connectedness		Safety		Work Design		Capability	
		M	SD	M	SD	M	SD	M	SD	M	SD
Health Care and Social Assistance	1396	68.5	20.8	78.4	13.7	74.5	18.8	65.1	21.4	66.9	20.3
Retail Trade	987	72.1	18.2	77.9	13.4	76.8	16.5	71.3	17.6	68.5	20.8
Professional, Scientific, and Technical Services	919	74.1	18.4	80.1	12.7	77.3	17.9	74.3	17.4	69.3	21.6
Construction	896	75.2	15.2	79.2	11.3	77.6	15.5	74.2	16.9	70.9	19.5
Education and Training	837	69.5	20.0	78.0	13.2	75.5	17.9	65.3	21.5	67.8	20.5
Manufacturing	700	77.0	13.6	79.7	10.3	80.1	12.9	76.2	15.1	75.2	17.3
Accommodation and Food Services	681	71.7	19.1	78.4	13.8	74.6	19.4	70.1	19.5	62.4	24.6
Public Administration and Safety	553	67.5	20.0	77.7	12.5	75.3	18.7	67.5	20.5	70.4	17.4
Transport, Postal, and Warehousing	522	72.8	20.0	78.6	13.5	76.7	19.0	71.8	20.2	71.8	19.6
Administrative and Support Services	379	72.3	21.7	79.1	13.6	76.7	18.8	71.9	19.8	68.7	22.9
Financial and Insurance Services	377	75.1	15.6	78.8	12.2	79.0	15.2	75.0	15.5	73.1	17.2
Wholesale Trade	304	73.0	16.2	77.0	11.3	76.1	17.0	73.5	15.3	69.1	21.5
Agriculture, Forestry and Fishing	215	75.8	16.6	79.7	12.3	77.9	15.6	76.2	16.3	70.3	21.9
Arts and Recreation Services	207	75.4	16.9	81.3	12.0	78.4	17.2	73.2	17.4	69.9	19.9
Information Media and Telecommunications	187	76.3	13.2	79.2	10.8	80.7	13.9	76.6	13.5	74.0	16.9
Mining	186	69.0	22.5	77.9	13.8	79.0	20.2	69.9	20.5	71.0	20.1
Rental, Hiring, and Real Estate Services	175	74.5	16.6	79.8	12.0	74.7	19.0	73.5	19.2	66.4	22.7
Electricity, Gas, Water, and Waste Services	145	76.0	16.6	79.4	12.6	78.5	16.5	76.0	17.3	73.7	18.3
Other Services	264	70.3	17.8	78.2	13.0	72.6	19.4	67.9	18.8	59.9	21.4
ANOVA analysis results											
df		9922		9929		9919		9929		9924	
F		13.35		2.62		6.18		23.83		14.90	
p		< 0.001		< 0.001		< 0.001		< 0.001		< 0.001	
Partial eta		0.024		0.005		0.11		0.041		0.026	

Scores for each industry calculated according to formula (average of domain items −1)*25. Scores are means (M) and standard deviations (SD)

## Discussion

This study has outlined the development of a valid set of five domains of a mentally healthy, thriving workplace to enable meaningful comparisons across industries and sectors. From over one hundred workplace items, five distinct domains emerged from and were confirmed by the analyses. Further, the emerging domains of Leadership, Connectedness, Safety, Work Design, and Capability demonstrated small but significant differences across the 19 ANZSIC industries represented by the worker respondents. This analysis is the first step in establishing the domains as a measurement tool for industry-specific quantification of workplace well-being factors, with additional potential for their application as a basis of evaluation of the effectiveness of interventions to address workplace psychosocial risk factors.

The five domains identified in the current study align with the integrated approach to workplace mental health [19] that is central to the three overarching strategies of the joint International Labour Organization (ILO) and WHO policy brief on Mental Health at Work launched in late September, 2022 [30]. The three pillars of the integrated approach [19, 31] are represented across items from within the five domains with the ‘protect’ pillar (i.e. identification of work-related hazards, protective effect of management support and compassionate leadership [32, 33]) corresponding to the Safety and Leadership domains; the ‘promote’ pillar (enhancing the positive aspects of work, such as meaningful social interaction and flexible work arrangements) reflected in items particularly within the Connectedness and Work Design domains; and the ‘respond’ pillar (resources available to employees experiencing distress [11]) aligning with items within the Capability and Safety domains. Whilst mapping of the items representing the five domains across these three pillars is beyond the scope of the current study, the wording of the items was developed to ensure strong representation of each of these pillars within the five domains. This ensures that the emerging actionable insights from the ITW are couched within key domains that *protect* workers from harm, *promote* positive behaviours, and *provide support* (respond) where required.

These domains of a thriving workplace therefore position the ITW as a comprehensive tool that can address the integrated approach in its entirety. A recent review identified only one existing tool developed in the Australian context that operated across all three pillars, the FlourishDX tool [31]. A key differentiating factor for the ITW Domains framework is its evolution since 2015, more specifically its foundation in data emerging from the largest national workplace mental health survey in Australia, even providing possibilities to describe historical changes at an industry level up to a decade ago. With further testing there is also potential that the domains could be used to inform organisations about the extent to which they align with key recommendations for employers put forward by the ILO to enable prevention of psychosocial hazards and risks in the workplace. Of particular relevance are the recent recommendations around increasing employee control over work tasks, allowing workers to have greater participation in decision-making (captured by the Work Design Domain) and building social support systems for workers within the workplace (Connectedness Domain) [34].

Historically, measurement tools designed to tap into psychosocial risk and/or mental health and well-being in the workplace with the intent to inform and enable better management of workplace mental health and well-being have largely taken a compliance approach focussing primarily on controlling exposure to psychosocial hazards. The domains emerging from the current study are unique in that they take a positive approach to workplace mental health by differentiating between markers of *thriving in the workplace*; this has been done in the context of the US working population [10] but not yet for the Australian workforce. The associated strength-based approach applied to the item wording within each domain serves to identify areas of positive action and perceptions in relation to workplace [35, 36]. The validation of the domains therefore positions the Indicators of a Thriving Workplace (ITW) questionnaire as the first comprehensive measure of workplace mental health and well-being that focuses on *thriving* as a positive construct for Australian workers.

This study has established the existence of industry-based differences across the domains of a thriving workplace, suggesting that intervention approaches should be matched to the specific needs of each industry to maximise the impact. Adding an industry-level layer to what is known in this field provides an avenue for developing tailored intervention strategies that are responsive to the unique needs of organisations [31] and a method of targeting industries experiencing the highest rates of claims for compensation [4]. Considering serious mental health claims alone, the Healthcare and Social Assistance industry has one of the highest rates of claims reported in the most recently available WHS claims data (2021–22) [6]. The findings from the current analysis suggest that improving domains of Leadership and Work Design for Healthcare (68.5 and 65.1, respectively) should be a priority as these are some of the lowest scores across all industries, and may impact the pattern of future claims. A further example of the utility of the domains approach is to identify areas of strength from particular industries that may be translatable to other less well-performing industries. For example, what is contributing to the high performance in Safety and Capability within Manufacturing (domain scores of 80.1 and 75.2, respectively), and how can other industries learn and adapt what they are doing to improve in these areas? By highlighting industry differences, domain profiling also enables a shift away from interventions directed towards individual workers and illnesses towards industry-wide initiatives; a shift that is critical if we are to see measurable positive change in working conditions and the mental health of Australian employees [16].

To positively impact work-related mental health outcomes, efforts in this space need to be evidence-informed, targeted, and actionable and preferably target organisation-wide working conditions as opposed to the individual worker. The largest variation in the domains across industries was seen for Work Design. Current opinion suggests that variability across work design may be driven by current global workforce trends such as increasing diversity and the industry-specific susceptibility to changes (such as job insecurity) due to advances in artificial intelligence, automation, and robotisation (i.e. ‘Industry 4.0’) [37]. *Prevention* efforts addressing challenges relating to the Work Design domain will, by necessity, be highly industry and context specific and will require varied and individualised approaches [38–40]; interventions focussing on increasing autonomy and/or customising work scheduling have some success, but efficacy is context dependent [40]. It may be that the application of more generalised approaches to work design challenges has contributed to the mixed evidence in favour of interventions in the area of job control [16].

In contrast, interventions falling under the *promotion* and *respond* action areas such as targeting manager/supervisor awareness and skills around identifying early signs of mental ill-health have stronger evidence supporting their effectiveness in improving worker mental health outcomes [16]. Such efforts focussing on the domains of Leadership and Capability are likely to share common elements explaining in part the smaller variations across industry domain scores. An example of this is evidence that compassionate leadership (through training and education) can help to mitigate burnout, improve retention, and promote well-being in healthcare and more broadly in education, public service, and business sectors [41, 42].

The current study’s results suggest that approaches to addressing workplace mental health are likely to be more successful when they are industry specific and for some interventions more than others. Interventions *responding* to mental health challenges in the workplace (third pillar) such as anti-stigma interventions known to encourage disclosure of mental health and improve inclusivity [16] may require less cross-industry tailoring to enable positive outcomes. In contrast, efforts aligning with the *prevention* and *promote* action areas are perhaps more likely to require tailoring to effectively address key differences in domain profiles across industries, organisations, departments, divisions, and even roles. Further research is required to determine whether this is indeed the case.

### Areas for Future Research

Future research will further establish the validity and reliability of the domains framework in other samples of Australian workers. The five domains of a thriving workplace including Leadership, Connectedness, Safety, Work Design and Capability provide a number of clear future research opportunities to maximise thriving in the workplace for all Australian workers. Learning from industries and organisations doing well in this space, and adapting best practice psychosocial policies and practices is one area that was identified above. Research could also be directed towards vulnerable cohorts of workers to determine the strengths and weaknesses across their domain profiles, informing targeted workplace interventions to better support their mental health and well-being. Further, establishing domain profiles for different segments of our workforce, such as workers of a particular generation or culture, could allow us to be more strategic and leverage the strength of our increasingly diverse workforce to improve mental health and work outcomes across Australia.

Finally, surveying at an organisational level will enable further in-depth exploration within industries to pinpoint particular segment, departmental, and work role characteristics that are contributing to either favourable or poor performance within particular domains. This level of detail will allow for further opportunities for cross-industry adoption, and adaptation where required, of best practices.

### Strengths and Limitations

A strength of the methods applied within this study is the separation of a large pool of respondents into a development and confirmation sample. The domains derived from the initial sample were confirmed, providing a high level of confidence that the domains are valid indicators of workplace mental health and well-being. The items that form each domain are conceptually aligned, and correspond with components of existing theoretical frameworks of workplace mental health. Drawn from workplace psychosocial factors known to influence worker outcomes, the five domains provide a straightforward explanation of key workplace mental health concepts and a scoring method that permits rapid interpretation and identification of priority areas for intervention to improve a working environment. When captured regularly (e.g. annually) across a large, nationally representative sample of workers, these domains provide a meaningful, informative summary or overview of workplace mental health across Australia; a current snapshot that permits ongoing monitoring over time.

Limitations of the current study include the method of data collection and the potential bias in using data collection agency panels to recruit survey participants. It is possible that the people responding to the survey are not truly representative of the Australian working population. Efforts to minimise the impact of this bias include the recruitment of a large number of participants, application of population-based quotas and data weighting according to population-level information. Another limitation is the identification of a potential 11-factor solution within the initial analysis; indeed, this model is likely to explain a greater level of variance in the data than the developed 5-factor solution. However, there are clear benefits in the relative simplicity of communicating a set of 5 domain scores instead of 11, which also translates to a more effective tool for quantifying key components of workplace mental health and well-being. Finally, our industry comparisons are dependent on the accuracy of survey respondents self-selecting their working industry based on the 19 defined by ANZSIC [22]. The reliability of self-selection of industry should be investigated in the future applications of the domains.

## Concluding Remarks

From more than 100 indicators of workplace mental health and well-being, the five domains of Leadership, Connectedness, Safety, Work Design, and Capability emerged from our primary factor analysis and were subsequently confirmed. When average domain scores were compared across industries, small but statistically significant differences were identified. Industry-based domain profiling could provide a basis for the prioritisation of efforts to improve workplace mental health and identify successful initiatives and practices that could be adapted to other industries. The established domains of a thriving workplace are a readily understandable method of communication and quantification of key workplace factors that influence worker mental health and well-being.

## Data Availability

Data from the Indicators of a Thriving Workplace annual survey are owned by SuperFriend and can be made available on request.

## Appendix A

Items of each domain and associated component loadings for the confirmation sample.

**Table:**

	1	2	3	4	5
	Leadership	Connectedness	Safety	Work Design	Capability
Cronbach’s alpha coefficient	0.93	0.86	0.92	0.91	0.89
Item component loadings					
Managers demonstrate the values of the workplace​	0.65				
Managers advocate for the best interests of their team​	0.66				
Managers support their team to work together​	0.66				
Managers provide constructive feedback​	0.67				
Managers are available when people need them​	0.63				
Managers genuinely consider other people's ideas	0.69				
Managers match tasks with people’s strengths​	0.64				
Managers help their teams to solve problems​	0.67				
Managers make sure people have the resources they need to do their job well	0.60				
Managers demonstrate how they support their own mental health​	0.53				
I treat others with respect​		0.67			
I appreciate others whose backgrounds are different from my own​		0.62			
I am honest with others​		0.62			
I feel that I approach situations with an open mind​		0.55			
People care about each other​		**0.45**			
I trust the people with whom I work​		**0.41**			
I feel like I am part of a team​		**0.47**			
I feel that people use appropriate humour​		**0.48**			
People are polite to each other​		0.51			
I feel that people work together effectively​		**0.44**			
There are processes to minimise harassment​			0.72		
There is an effective process to investigate reports of harassment​			0.68		
There is a zero tolerance approach to harassment​			0.78		
There are processes to minimise bullying​			0.67		
There is an effective process to investigate reports of bullying​			0.64		
There is a zero tolerance approach to bullying​			0.77		
There are processes to minimise discrimination​			0.65		
There is an effective process to investigate reports of discrimination​			0.67		
There is a zero tolerance approach to discrimination​			0.74		
There is support available if violence is experienced at work​			0.52		
Managers support the practice of good work-life balance​				0.63	
My workplace recognises work-life balance looks different for everyone				0.71	
My workplace supports flexible working arrangements				0.82	
My workplace provides equal access to flexible work arrangements regardless of individual circumstances				0.73	
People at my workplace have enough time to rest between scheduled work times​				0.60	
People at my workplace can work at times that best suit them				0.83	
People at my workplace have enough breaks whilst they’re at work to recharge				0.60	
People at my workplace are given flexibility to control when they complete work tasks				0.66	
People at my workplace are given flexibility to control how they complete work tasks				0.57	
People at my workplace can contribute to decisions that affect their job				**0.48**	
There is access to confidential counselling or support services​					0.77
There are no barriers to accessing confidential counselling or support service​					0.69
In general, people in my workplace have the skills and expertise to support each other’s mental health​					**0.49**
In general, people in my workplace have access to mental health education​					0.71
Managers participate in regular mental health training​					0.60
There is support for training initiatives aimed at improving human resilience​					**0.47**
There is adequate training in how to respond to traumatic events at work​					**0.48**

Items in bold are eigenvalues below the 0.5 threshold but remained the strongest loading items in that domain and were retained.

## References

[1] Chang R. The impact of employees’ health and well-being on job performance. J Edu Human Soc Sci. 2024;29:372–8. 10.54097/9ft7db35.

[2] Sorensen G, Dennerlein JT, Peters SE, Sabbath EL, Kelly EL, Wagner GR. The future of research on work, safety, health and wellbeing: a guiding conceptual framework. Soc Sci Med. 2021;269:113593. 10.1016/j.socscimed.2020.113593. 33341740 10.1016/j.socscimed.2020.113593 PMC10868656

[3] Department of Health and Aged Care. National mental health workforce strategy: 2022-2032. Australian Government; 2022. p. 1–67.

[4] Safe Work Australia. Australian Work Health and Safety (WHS) Strategy 2023–2033. 2023.

[5] Committee for Economic Development Australia (CEDA). Mental health and the workplace: how can employers improve productivity through wellbeing? CEDA. 2022.

[6] Safe Work Australia. Key work health and safety statistics Australia 2022. 2023.

[7] Safe Work Australia. Psychological health and safety in the workplace. Safe Work Australia. 2024.

[8] Dollard MF, Hall G, LaMontagne AD, Taylor AW, Winefield AH, Smith P, et al. Australian Workplace Barometer (AWBQ2009). University of South Australia. 2009.

[9] Dollard MF, Bailey TS, Hall G. Surveillance system for psychosocial risk and testing the Australian workplace barometer theoretical model. The Australian Workplace Barometer: psychosocial safety climate and working conditions in Australia. Samford Valley, QLD: Australian Academic Press. 2014.

[10] Peters SE, Gundersen DA, Katz JN, Sorensen G, Wagner GR. Thriving from work questionnaire: dimensionality, reliability, and validity of the long and short form questionnaires. Am J Ind Med. 2023;66(4):281–96. 10.1002/ajim.23465. 36748853 10.1002/ajim.23465 PMC13048217

[11] Deady M, Sanatkar S, Tan L, Glozier N, Gayed A, Petrie K, et al. A mentally healthy framework to guide employers and policy makers. Front Public Health. 2024;12:1430540. 10.3389/fpubh.2024.1430540. 39109149 10.3389/fpubh.2024.1430540 PMC11301648

[12] Safe Work Australia. Managing psychosocial hazards at work. Safe Work Australia. 2022. p. 54.

[13] Mental Health Commission of Canada. The national standard of Canada for psychological health and safety in the workplace. Mental Health Commission of Canada. 2013.

[14] SafeWork NSW. Code of practice: managing psychosocial hazards at work. NSW Government. 2021. p. 7.

[15] WorkSafe Victoria. A guide for employers: Preventing and managing work-related stress. 2021. p. 54.

[16] Rugulies R, Aust B, Greiner BA, Arensman E, Kawakami N, LaMontagne AD, et al. Work-related causes of mental health conditions and interventions for their improvement in workplaces. Lancet. 2023;402(10410):1368–81. 10.1016/S0140-6736(23)00869-3. 37838442 10.1016/S0140-6736(23)00869-3

[17] Andersen JH, Malmros P, Ebbehoej NE, Flachs EM, Bengtsen E, Bonde JP. Systematic literature review on the effects of occupational safety and health (OSH) interventions at the workplace. Scand J Work Environ Health. 2019;45(2):103–13. 10.5271/sjweh.3775. 30370910 10.5271/sjweh.3775

[18] Bondebjerg A, Filges T, Pejtersen JH, Kildemoes MW, Burr H, Hasle P, et al. Occupational health and safety regulatory interventions to improve the work environment: an evidence and gap map of effectiveness studies. Campbell Syst Rev. 2023;19(4): e1371. 10.1002/cl2.1371. 38089568 10.1002/cl2.1371 PMC10712440

[19] LaMontagne AD, Shann C, Martin A. Developing an integrated approach to workplace mental health: a hypothetical conversation with a small business owner. Ann Work Exp Health. 2018;62(Supplement_1):S93–100. 10.1093/annweh/wxy039. 10.1093/annweh/wxy039 30212883

[20] 2024. https://www.abs.gov.au/statistics/labour/employment-and-unemployment/labour-force-australia. Accessed 2/8/2024.

[21] Wu A, Roemer EC, Kent KB, Ballard DW, Goetzel RZ. Organizational best practices supporting mental health in the workplace. J Occup Environ Med 2021;63(12):e925–e931. 10.1097/JOM.0000000000002407 PMC8631150 34840320

[22] Australian Bureau of Statistics: Australian and New Zealand Standard Industrial Classification (ANZSIC). https://www.abs.gov.au/statistics/classifications/australian-and-new-zealand-standard-industrial-classification-anzsic/latest-release (2006). Accessed.

[23] Pallant J. SPSS Survival Manual: A step by step guide to data analysis using IBM SPSS. 7th ed. London: Routledge; 2020.

[24] Tabachnick BG, Fidell, L.S. Using multivariate statistics. 6th ed. Pearson Education. 2013.

[25] Horn JL. A rationale and test for the number of factors in factor analysis. Psychometrika. 1965;30(2):179–85. 10.1007/BF02289447. 14306381 10.1007/BF02289447

[26] Hair JF, Black WC, Babin BJ, Anderson RE. Multivariate data analysis. Essex, UK: Pearson Education Limited. 2014.

[27] Thompson B. Exploratory factor analysis decision sequence. Exploratory and confirmatory factor analysis: understanding concepts and applications. Washington, DC, US: American Psychological Association. 2004. p. 27–48.

[28] Pett MA, Lackey NR, Sullivan JJ. Making sense of factor analysis. SAGE Publications Inc.; 2003.

[29] Cohen J. Statistical power analysis for the behavioral sciences 2nd Ed ed. Hillsdale, NJ: Lawrence Earlbaum Associates. 1988.

[30] World Health Organization and International Labour Organization. Mental health at work: policy brief. In: World Health Organization, editor. 2022. p. 20.

[31] Nebbs A, Martin A, Neil A, Dawkins S, Roydhouse J. An integrated approach to workplace mental health: a scoping review of instruments that can assist organizations with implementation. Int J Environ Res Public Health. 2023. 10.3390/ijerph20021192. 36673948 10.3390/ijerph20021192 PMC9859114

[32] Gayed A, Milligan-Saville JS, Nicholas J, Bryan BT, LaMontagne AD, Milner A, et al. Effectiveness of training workplace managers to understand and support the mental health needs of employees: a systematic review and meta-analysis. Occup Environ Med. 2018;75(6):462–70. 10.1136/oemed-2017-104789. 29563195 10.1136/oemed-2017-104789

[33] Petrie K, Gayed A, Bryan BT, Deady M, Madan I, Savic A, et al. The importance of manager support for the mental health and well-being of ambulance personnel. PLoS One. 2018;13(5):e0197802. 10.1371/journal.pone.0197802. 29791510 10.1371/journal.pone.0197802 PMC5965892

[34] International Labour Organization: Psychosocial risks and stress at work. https://www.ilo.org/resource/psychosocial-risks-and-stress-work (2022). Accessed 5th September 2024.

[35] LaMontagne AD, Martin A, Page KM, Reavley NJ, Noblet AJ, Milner AJ, et al. Developing an integrated approach to workplace mental health. Total worker health. Washington, DC, US: American Psychological Association; 2019. p. 211–27.

[36] LaMontagne AD, Martin A, Page KM, Reavley NJ, Noblet AJ, Milner AJ, et al. Workplace mental health: developing an integrated intervention approach. BMC Psychiatry. 2014;14:131. 10.1186/1471-244x-14-131. 24884425 10.1186/1471-244X-14-131 PMC4024273

[37] Fraccaroli F, Zaniboni S, Truxillo DM. Challenges in the new economy: a new era for work design. Ann Rev Organ Psychol Organ Behav. 2024;11:307–35. 10.1146/annurev-orgpsych-081722-053704.

[38] Ciarlante K, Mejia C, Broker E. A research agenda for occupational safety, health, & well-being in hospitality & tourism management. Int J Hosp Manag. 2024;123:103887. 10.1016/j.ijhm.2024.103887.

[39] Knight C, Parker SK. How work redesign interventions affect performance: an evidence-based model from a systematic review. Human Relations. 2021;74(1):69–104. 10.1177/0018726719865604.

[40] Dettmers J, Bredehöft F. The ambivalence of job autonomy and the role of job design demands. Scand J Work Organ Psychol. 2020. 10.16993/sjwop.81.

[41] Östergård K, Kuha S, Kanste O. Health-care leaders’ and professionals’ experiences and perceptions of compassionate leadership: a mixed-methods systematic review. Leadersh Health Serv. 2024;37(5):49–65. 10.1108/LHS-06-2023-0043. 10.1108/LHS-06-2023-0043 PMC10868663 37823549

[42] Ramachandran S, Balasubramanian S, James WF, Al MT. Whither compassionate leadership? Syste Rev Manag Rev Quart. 2024;74(3):1473–557. 10.1007/s11301-023-00340-w.

